# Outcomes and prognostic factors of non-HIV patients with pneumocystis jirovecii pneumonia and pulmonary CMV co-infection: A Retrospective Cohort Study

**DOI:** 10.1186/s12879-017-2492-8

**Published:** 2017-06-05

**Authors:** Qing Yu, Peng Jia, Li Su, Hong Zhao, Chengli Que

**Affiliations:** 1 Department of Respiratory and Critical Care Medicine, Peking University First Hospital, Beijing, 100034 China; 2 Department of Infectious Disease, Peking University First Hospital, Beijing, 100034 China

**Keywords:** *Pneumocystis jirovecii*pneumonia, Cytomegalovirus, CMVDNA load, Prognosis

## Abstract

**Background:**

*Pneumocystis jirovecii* pneumonia (PJP) and pulmonary cytomegalovirus (CMV) infection are common opportunistic infections among immunocompromised patients. However, few studies have evaluated their co-infection, especially among non-HIV patients. Therefore, we aimed to evaluate the outcomes and prognostic factors among non-HIV patients with PJP according to their CMV infection status.

**Methods:**

This retrospective study evaluated non-HIV patients who were diagnosed with PJP between January 2009 and January2016.The patients were classified and compared according to their pulmonary CMV infection status (positive infection: bronchoalveolar lavage fluid [BALF] CMV DNA loads of >500copies/mL).

**Results:**

Among 70 non-HIV patients with PJP, we identified 38 patients (54.3%) with pulmonary CMV infection. There was no significant difference in the mortality rates for the two groups (*p* = 0.15). Pulmonary CMV infection was significantly more common among patients who were receiving glucocorticoids and immunosuppressants, compared to corticosteroids only (*p* = 0.02). Pulmonary CMV infection was also significantly associated with severe dyspnea, a lower PaO_2_/FiO_2_, and the presence of centrilobular nodules (*p* = 0.008). Higher CMV DNA loads in the BALF were positively associated with mortality (*p* = 0.012).

**Conclusions:**

Combined therapy using corticosteroids and other immunosuppressants may be a risk factor for pulmonary CMV co-infection among patients with PJP. In addition, CMV pneumonia should be considered when centrilobular nodules and/or severe hypoxemia are observed in non-HIV patients with PJP. Furthermore, antiviral treatment should be promptly initiated for patients with a high CMV DNA load in BALF, based on their poor prognosis.

## Background

*Pneumocystis jirovecii* pneumonia (PJP) is a common opportunistic infection among immunocompromised patients, and especially among patients with human immunodeficiency virus (HIV) infection. In addition, autoimmune diseases, malignancy, organ transplantation, and treatment using immunosuppressants and antineoplastic drugs increase the risk of *P. jirovecii* infection [1–5]. Cytomegalovirus (CMV) infection is another common cause of pneumonia in immunocompromised hosts, and CMV co-infection can be observed in patients who are infected with other pathogens (especially *P. jirovecii*) [6]. Previous studies have reported that 28–69% of HIV-infected patients also exhibit co-infection with *P. jirovecii*andCMV [7–10]. However, CMV can also be found in the respiratory secretions of patients without clear signs of pneumonia [11], and the pathogenesis and prognosis of pulmonary CMV infection with PJP in non-HIV patients is poorly understood.

In an animal model, CMV altered the host’s immune environment by modulating molecules that are involved in immune recognition and inflammation [12]. Moreover, CMV infection can alter the host’s immune response by suppressing the function of helper T-cells and antigen-presenting cells, which are essential for achieving PJP resolution [12]. Nevertheless, researchers have not found any significant differences in the morbidity and mortality rates among HIV-infected patients with PJP according to their pulmonary CMV infection status [7, 9, 13]. Although the clinical characteristics and prognosis of PJP are different in HIV-infected and non-HIV patients [14, 15], there are few reports regarding PJP and CMV co-infection among non-HIV patients, despite a reported incidence of 29.2% (31/106) in a study by Tark et al. [13].

The present study aimed to evaluate the outcomes and prognostic factors among non-HIV patients with PJP according to their pulmonary CMV infection status.

## Methods

### Patients

For this study, we retrospectively identified non-HIV patients who were diagnosed with PJP between January 2009 and January 2016. These patients had undergone bronchoalveolar lavage using a flexible bronchoscope and the standard techniques [16]. The bronchoalveolar lavage fluid (BALF) specimens were processed and stained using Gomori methenamine silver (GMS), and the BALF specimens were also evaluated for the presence of pneumocystis cysts. The results of these evaluations and the patient’s clinical manifestations were used to confirm a diagnosis of PJP. In this study, we also compared the demographic characteristics, clinical characteristics, and rates of morbidity and mortality among non-HIV patients with PJP according to their pulmonary CMV infection status.

### Microbiological methods

The BALF specimens were also sent to microbiological laboratory using Gram staining, acid-fast staining, and culture for bacterial and fungal infection. Real-time quantitative polymerase chain reaction (PCR) was used to evaluate the BALF loads of CMV DNA for all patients, and a cut-off value of ≥500copies/mL(about 125 IU/ml) was considered positively identify pulmonary CMV infection, based on the manufacturer’s instructions (Human Cytomegalovirus Fluorescence Quantitative Polymerase Chain Reaction Diagnostic Kit; Da An Gene Company, Guangzhou, China).

### Statistical analysis

All analyses were performed using SPSS software (version 19.0; SPSS Inc., Chicago, IL). Categorical variables were compared using the chi-square test or Fisher’s exact test. Continuous variables were analyzed by median IQR and compare them using the Mann–Whitney U-test. The rank sum test was used to analyse the association between mortality and the BALF loads of CMV DNA. Differences with a *p*-value of <0.05 were considered statistically significant.

## Results

During the study period, we identified 70 non-HIV patients who were diagnosed with PJP (43 men, 27 women; median age: 50 years, range: 21–81 years). And all patients with PJP had undergone BALF testing for CMV-DNA. Thirty-eight patients (54.3%) exhibited pulmonary CMV infection (CMV DNA loads of ≥500copies/mL in their BALF). Twenty-eight patients exhibited bacterial co-infection and nineteen patients exhibited fungal co-infection. In our study, the PJP patients co-infection with CMV was significantly associated with combined use of glucocorticoids and T-cell immunosuppressants (*p* = 0.02). There was no significant difference in the mortality rates when we compared the patients with and without pulmonary CMV infection(17/38 [44.7%] vs.9/32 [28.2%], respectively; *p* = 0.15)(Table 1).

**Table 1:** The demographic characteristics, underlying diseases, and mortality rates among non-HIV patients with PJP according to pulmonary CMV infection status

	CMV-DNA positive in BALF (*n* = 38)	CMV-DNA negative in BALF(*n* = 32)	*p*
Demographics			
Age, yr., median	51(21–77)	48(22–81)	0.48
Gender ratio(male:female)	23:15	20:12	0.87
Underlying diseases,n(%)			
Solid organ transplantation	3(7.9)	3(9.4)	1.00
Hematologic malignancy	4(10.5)	9(28.2)	0.06
Hematopoietic stem cell transplantation	3(75)	4(44.4)	
Non-hematologic malignancy	2(5.3)	2(6.3)	1.00
Dermatologic diseases	4(10.5)	5(15.6)	0.78
Kidney disease	12(31.5)	5(15.6)	0.12
Interstitial lung disease	0(0)	1(3.1)	1.00
Connective tissue disease	9(23.7)	5(15.6)	0.40
Idiopathic thrombocytopenia	2(5.3)	1(3.1)	1.00
Others	2(5.3)	1(3.1)	1.00
Immunosuppressive agents,n(%)			
Steroids	37(97.4)	31(96.9)	1.00
Dosage of steroid, recent 1 month(mg)	40(10–100)	45(10–300)	0.54
Steroid + T-cell immunosuppressants	28(73.7)	15(65.2)	0.02
Steroid +Anti-CD20+ monoclonal antibody^a^	1(2.6)	6(18.8)	0.06
Chemotherapy/Radiotherapy	2(5.3)	3(9.4)	0.84
Treatment for PJP			
TMP-SMZ(%)	33(86.8)	31(96.9)	0.29
Treatment duration, days, median	15(4–31)	21(2–23)	0.051
Shift to second-line therapy^b^(%)	23(60.5)	12(37.5)	0.055
Due to treatment failure	18(47.4)	11(34.4)	0.27
Due to adverse reaction^c^	5(13.2)	1(3.1)	0.29
Morbidity,n(%)			
Admitted to ICU	16(42.1)	9(28.2)	0.22
Mechanical ventilation	14(36.8)	8(25.0)	0.29
Mortality	17(44.7)	9(28.2)	0.15

^a^Rituximab

^b^second-line therapy: primaquine plus clindamycin and caspofungin

^c^Adverse reactions included thrombocytopenia, bone marrow suppression, and drug allergy (2 cases)

*PJP Pneumocystis jirovecii* pneumonia, *CMV* cytomegalovirus, *BALF* bronchoalveolar lavage fluid, *TMP-SMZ* sulfamethoxazole and trimethoprim, *ICU* intensive care unit

In terms of C-reactive protein levels, CD4 + T-lymphocyte count, and lactate dehydrogenase levels, no significant differences were found between patients co-infected with or without CMV. However, patients with both PJP and CMV co-infection exhibited a significantly higher frequency of having a PaO_2_/FiO_2_of < 100(*p* = 0.04) (Table 2). We also analysed the patients’ clinical and radiographic manifestations (Table 3). Fever (84.3%) and dyspnea (65.7%) were the two most common symptoms, and patients with PJP and CMV co-infection exhibited a significantly higher rate of dyspnea (*p* = 0.04). Ground-glass opacities were observed during the chest computed tomography (CT) for all patients, with upper lobe predominance in15 cases and consolidation in 33 cases. However, there were no association between the lesion’s location or consolidation and pulmonary CMV infection (both, *p* > 0.05). Centrilobular nodules were identified on the CT scan in17 patients, and 14 of these patients had pulmonary CMV infection; this association was statistically significant (*p* = 0.008).

**Table 2:** The laboratory test results according to pulmonary CMV infection status

	CMV-DNA positive in BALF	CMV-DNA negative in BALF	*p*
WBC count (10^9^/L)	7.67 ± 3.56	10.16 ± 5.51	0.08
ALT elevation, n (%)	15(39.5%)	6(18.8%)	0.06
CRP, mg/L	31.1(0.21–266)	69.1(5.18–314)	0.24
LDH, IU/L	400(151–1167)	398(181–1918)	0.79
CD4 + T lymphocyte count,/ul	223.0 ± 171.6	319.4 ± 277.9	0.13
Neutrophils in BALF, %	33(0–96)	17(3–97)	0.41
Lymphocyte in BALF,%	17(0–76)	32(0–80)	0.12
Pulmonary co-infection, n (%)			
Bacteria	18 (47.4%)	10 (31.3%)	0.17
Fungus	12 (31.6%)	7 (21.9%)	0.36
PaO2/FiO2,n(%)			
PaO2/FiO2 > 300	8(21.1%)	11 (34.4%)	0.21
200 < PaO2/FiO2 ≤ 300	10 (26.3%)	9 (28.1%)	0.86
100 < PaO2/FiO2 ≤ 200	7 (18.4%)	8 (25.0%)	0.50
PaO2/FiO2 ≤ 100	13(34.2%)	4 (12.5%)	0.04

*CMV* cytomegalovirus, *BALF* bronchoalveolar lavage fluid, *WBC* white blood cell, *ALT* alanine aminotransferase, *CRP* C-reactive protein, *LDH* lactate dehydrogenase

**Table 3:** The patients’ clinical and radiographic manifestations according to pulmonary CMV infection status

	CMV-DNA positive in BALF	CMV-DNA negative in BALF	*p*
Symptoms, n (%)			
fever	31 (81.6%)	28 (87.5%)	0.50
dyspnea	29 (76.3%)	17 (53.1%)	0.04
CT findings			
ground-glass opacities	38/38 (100.0%)	32/32 (100.0%)	1.00
consolidations	18/38 (47.4%)	15/32 (46.9%)	0.97
centrilobular nodules	14/38 (36.8%)	3/32 (9.4%)	0.008
upper lobe predominance	7/38 (18.4%)	8/32 (25.0%)	0.50

*CMV* cytomegalovirus, *BALF* bronchoalveolar lavage fluid, *CT* computed tomography

We also measured the serum loads of CMV DNA and found that 21 patients had detectable CMV DNA in their serum and BALF specimens. Seventeen patients exhibited positive BALF specimens, but negative serum specimens. None of the 32 patients with negative BALF specimens exhibited detectable CMV DNA in their serum. We observed a positive association between BALF loads of CMV DNA and mortality among the 38 patients with PJP and pulmonary CMV co-infection (Table 4).

**Table 4:** The association between mortality and CMV DNA in bronchoalveolar lavage fluid

CMV-DNA load in BALF(copies/ml)	Prognosis		*p*
	survivor (n)	non-survivor(n)	
≤1 × 10^4^	10	3	0.012
1 × 10^4^ ~ 1 × 10^5^	6	2	
≥1 × 10^5^	5	12	

*CMV* cytomegalovirus, *BALF* bronchoalveolar lavage fluid

## Discussion

Among HIV-infected patients, PJP and pulmonary CMV co-infection is relatively common (28–69% of all cases) [7–10], and the CMV infection is often related to the patient’s non-specific immunosuppression. Although PJP is a relatively indolent process in HIV-infected patients, it is usually a potentially life-threatening infection among immunocompromised non-HIV patients. Thus, although there is no significant difference in mortality among HIV-infected patients with PJP according to their CMV infection status [7, 9, 13], we cannot extrapolate it to non-HIV counterparts.

In the present study, we observed that 54.3% of our non-HIV patients with PJP exhibited CMV co-infection, with subgroup analyses revealing co-infection rates of 31.5% among patients with kidney disease and 20% among patients with hematological malignancies who had not received hematopoietic stem cells (Table 1). Tark et al. attributed the different rates of CMV co-infection to the use of T-cell immunosuppressants, as they found that the use of these agents was significantly associated with CMV pneumonia [13]. Our findings support their conclusions, as CMV co-infection in the present study was significantly associated with the combined use of glucocorticoids and T-cell immunosuppressants (*p* = 0.02). We found the significant association between the use of T-cell immunosuppressants and the morbidity among patients with PJP and CMV co-infection (*p* = 0.02). Therefore, it appears that the use of T-cell immunosuppressants may be a risk factor for CMV co-infection in PJP patients (odds ratio [OR]: 3.32,95% confidence interval [CI]: 1.19–9.29). Furthermore, a PaO_2_/FiO_2_ of ≤100 was significantly more common among patients with PJP and pulmonary CMV co-infection(*p* = 0.035), which suggests that this form of co-infection causes more severe lung injuries, compared to PJP alone, in non-HIV patients. Therefore, when non-HIV patients with PJP present with severe hypoxemia, the possibility of pulmonary CMV co-infection should be excluded.

The initial symptoms of PJP are non-specific and including fever, dyspnea, cough, and ground-glass opacities on chest CT. If it is left untreated, PJP it will quickly progress to respiratory failure, especially among non-HIV patients [17]. In the present study, dyspnea was significantly more common among patients with PJP and CMV co-infection, compared to PJP alone (*p* = 0.04, odds ratio [OR]: 2.84,95% confidence interval [CI]: 1.03–7.89). Besides, patients with PJP and pulmonary CMV co-infection are more likely to exhibit lower PaO_2_/FiO_2_.

Among non-HIV patients with immunosuppression, the radiographical evidence of CMV pneumonia typically includes poorly-defined ground-glass opacities, small nodules, the tree-in-bud pattern, and the halo sign during high-resolution computed tomography (HRCT). These findings are predominantly distributed in the middle and lower lung fields, while thickening of the bronchovascular bundles and pleural effusion arerare [18, 19]. However, ground-glass opacities with an apical-predominant distribution, the mosaic pattern, the crazy-paving pattern, and cystic changes are common HRCT findings in cases of PJP [20–22]. In the present study, we found that PJP and CMV co-infection was significantly associated with the presence of centrilobular nodules (*p* = 0.008,OR: 5.64, 95%CI: 1.45–21.95), which indicates that CMV pneumonia should be considered in the differential diagnosis of non-HIV patients with PJP who exhibit centrilobular nodules.

CMV infection may manifest as a primary infection, latent infection, reactivated infection, or reinfection. The most common form in the general population is a latent infection, and patients with a latent infection may transmit the infection to other individuals through their bodily fluids. In addition, latent infections typically have no clinical effects unless the host becomes immunocompromised. As with other herpes viruses, latent CMV can be reactivated during periods of stress, and especially in immunocompromised adults with a critical illness [23]. Nichols et al. has found that the human CMV reactivation is common in these cases due to the immunocompromised state of patients [24]. And Peres et al. has reported that monitoring the CMV reactivation and preemptive or prophylactic treatment are critical for these patients [25]. The emergence of rapid PCR detection methods has facilitated the accurate, rapid, and quantitative detection of CMV DNA in the patient’s bodily fluids [26]. Furthermore, PCR can be used to detect CMV reactivation, although some researchers have reported that CMV reactivation could simply be an indicator of immunecompromise status and illness severity, and may not require diagnostic procedures and treatment [27]. Nevertheless, Yu et al. found that in the immunocompromised patients with rheumatic diseases can be diagnosed with CMV pneumonia based on serum CMV DNA loads of >1.75 × 10^4^copies/mL [28]. Moreover, Madi N et al. found in renal transplant patients with symptomatic CMV infection the CMV-DNA in serum were all more than 6.5 × 10^4^copies/mL. While at present, only a few studies have evaluated a cut-off value for CMV-DNA in BALF [29, 30]. Drew et al. has reported that CMV DNA levels of >5 × 10^5^copies/mL in the patient’s BALF confirmed the presence of CMV pneumonia [31], and Boeckh et al. found that CMV viral load > 500 IU/ml in BALF was likely to represent CMV pneumonia in hematopoietic stem cell transplantation patients [32]. Therefore, based on the absence of standardized CMV DNA assays, additional studies are needed to identify an accurate and reliable cut-off values for CMV DNA in BALF to identify CMV infection and pneumonia.

In our study, the 38 patients with pulmonary CMV co-infection received intravenous ganciclovir combined with anti-pneumocystis therapy, while the patients with only PJP received anti-pneumocystis treatment alone. There was no significant difference in mortality between these two groups (*p* = 0.15). There are two possible explanations for this phenomenon. Firstly, gancicloviris is considered effective for CMV infection. Secondly, the pulmonary CMV infection that we detected may represent CMV reactivation, rather than CMV pneumonia. Besides, in our patients with CMV-DNA positive in BALF, only 30% were found CMV-DNA positive in their serum, which indicates that the BALF testing provided greater sensitivity. Furthermore, the BALF-positive serum-negative patients exhibited CMV DNA loads of <1 × 10^4^copies/mL, which indicates that we cannot exclude the possibilities of local inflammation and/or CMV reactivation. In this context, some researchers have classified CMV infection according to the BALF viral load, with low-level infections referring to a load of <1 × 10^4^copies/mL, moderate infections having a load between 1 × 10^4^ to 1 × 10^5^copies/mL, and serious infections with a load of >1 × 10^5^copies/mL [33]. Moreover, Bauer et al. has confirmed that higher levels of CMV DNA were detectable in infected tissues [34]. In the present study, we used the above-mentioned grading system to classify the patients’ CMV infections and found that higher CMV DNA loads were significantly associated with mortality (*p* = 0.012). Therefore, timely antiviral treatment is likely needed to reduce the risk of mortality in cases that with high loads of CMV-DNA in their BALF.

There are several limitations to our study. First, patients with confirmed CMV infection require antiviral therapy, and patients without CMV infection do not require antiviral therapy, which makes it impossible to create a control group that is CMV-negative and receives antiviral therapy. This lack of a control group may limit our ability to conclusively comment on the association between CMV infection and prognosis. Second, BALF that is positive for CMVDNA could be attributed to latent infection, reactivation, or CMV pneumonia, and many of the patients exhibited severe hypoxemia, which precluded the use of lung biopsy to diagnose CMV pneumonia. Third, the small single-center sample of patients is prone to selection bias.

## Conclusions

Our study indicates that pulmonary CMV co-infection was significantly associated with the combined use of glucocorticoids and T-cell immunosuppressants among non-HIV patients with PJP. In addition, CMV pneumonia must be considered when centrilobular nodules and severe hypoxemia are present in non-HIV patients with PJP. Furthermore, high BALF loads of CMV DNA were positively associated with mortality, which indicates that antiviral treatment should be rapidly administered when these non-HIV patients exhibit PJP and CMV co-infection. Nevertheless, larger scale, well-designed prospective studies are needed to clarify the unanswered questions.

## Abbreviations

BALF: Bronchoalveolar lavage fluid
CI: Confidence interval
CMV: Cytomegalovirus
CT: Computed tomography
HIV: Human immunodeficiency virus
HRCT: High-resolution computed tomography
OR: Odds ratio
PCR: Polymerase chain reaction
PJP: *Pneumocystis jirovecii* pneumonia

## References

[1] Thomas CF, Limper AH (2004). Pneumocystis pneumonia. N Engl J med.

[2] Roblot F, Godet C, Le Moal G, Garo B, Faouzi Souala M, Dary M (2002). Analysis of underlying diseases and prognosis factors associated with Pneumocystis carinii pneumonia in immunocompromised HIV-negative patients. Eur J Clin Microbiol Infect Dis.

[3] Pagano L, Fianchi L, Mele L, Girmenia C, Offidani M, Ricci P (2002). Pneumocystis carinii pneumonia in patients with malignant haematological diseases: 10 years’ experience of infection in GIMEMA centres. Br J Hematol.

[4] Sepkowitz KA (2002). Opportunistic infections in patients with and patients without acquired immunodeficiency syndrome. Clin Infect Dis.

[5] Zahar JR, Robin M, Azoulay E, Fieux F, Nitenberg G, Schlemmer B (2002). Pneumocystis carinii pneumonia in critically ill patients with malignancy: a descriptive study. Clin Infect Dis.

[6] Salomon N, Perlman DC (1999). Cytomegalovirus pneumonia. Semin Respir Infect.

[7] Jacobson MA, Mills J, Rush J, Peiperl L, Seru V, Mohanty PK (1991). Morbidity and mortality of patients with AIDS and first-episode Pneumocystis carinii pneumonia unaffected by concomitant pulmonary cytomegalovirus infection. Am rev Respir Dis.

[8] Miles PR, Baughman RP, Linnemann CC (1990). Cytomegalovirus in the bronchoalveolar lavage fluid of patients with AIDS. Chest.

[9] Bozzette SA, Arcia J, Bartok AE, McGlynn LM, McCutchan JA, Richman DD (1992). Impact of Pneumocystis carinii and cytomegalovirus on the course and outcome of atypical pneumonia in advanced human immunodeficiency virus disease. J Infect Dis..

[10] Benfield TL, Helweg-Larsen J, Bang D, Junge J, Lundgren JD (2001). Prognostic markers of short-term mortality in AIDS-associated Pneumocystis carinii pneumonia. Chest.

[11] Angelici E, Contini C, Sebastiani G, Folgori F, Delia S, Serra P (1996). Cytomegalovirus in bronchoalveolar lavage specimens from patients with AIDS: comparison with antigenaemia and viraemia. J med Microbiol.

[12] Freeman RB (2009). The ‘indirect’ effects of cytomegalovirus infection. Am J Transplant.

[13] Kim T, Moon SM, Sung H, Kim MN, Kim SH, Chio SH (2012). Outcomes of non-HIV-infected patients with Pneumocystis pneumonia and concomitant pulmonary cytomegalovirus infection. Scand J Infect Dis.

[14] Ewig S, Bauer T, Schneider C, Pickenhain A, Pizzulli L, Loos U (1995). Clinical characteristics and outcome of Pneumocystis carinii pneumonia in HIV-infected and otherwise immunosuppressed patients. Eur Respir J.

[15] Mansharamani NG, Garland R, Delaney D, Koziel H (2000). Management and outcome patterns for adult Pneumocystis carinii pneumonia, 1985 to 1995: comparison of HIV-associated cases to other immunocompromised states. Chest.

[16] Klech H, Pohl W. Technical recommendations and guidelines for bronchoalveolar lavage (BAL). Report of the European Society of Pneumology Task Group. Eur Respir J. 1989;2(6):561–585. 2663535

[17] Ho M, Mandell GL (1995). Cytomegalovirus. Mandell, Douglas, and Benett’s principles and practice of infectious disease (vol 2).

[18] Franquet T, Lee KS, Müller NL (2003). Thin-section CT findings in 32 immunocompromised patients with cytomegalovirus pneumonia who do not have AIDS. AJR am J Roentgenol.

[19] Gasparetto EL, Ono SE, Escuissato D, Marchiori E, Roldan L, Marques HL (2004). Cytomegalovirus pneumonia after bone marrow transplantation: high resolution CT findings. Br J Radiol.

[20] Webb WR, Müller NL, Naidich DP (2001). High-resolution CT of the lung.

[21] Boiselle PM, Crans CA, Kaplan MA (1999). The changing face of Pneumocystis carinii pneumonia in AIDS patients. AJR am J Roentgenol.

[22] Kuhlman JE, Kavuru M, Fishman EK, Siegelman SS (1990). Pneumocystis carinii pneumonia: spectrum of parenchymal CT findings. Radiology.

[23] La Rosa C, Diamond DJ (2012). The immune response to human CMV. Future Virol.

[24] Nichols WG, Corey L, Gooley T, Davis C, Boeckh M (2002). High risk of death due to bacterial and fungal infection among cytomegalovirus (CMV)-seronegative recipients of stem cell transplants from seropositive donors: evidence for indirect effects of primary CMV infection. J Infect Dis..

[25] Peres RM, Costa CR, Andrade PD, Bonon SH, Albuquerque DM, de Oliveria C (2010). Surveillance of active human cytomegalovirus infection in hematopoietic stem cell transplantation (HLA sibling identical donor): search for optimal cutoff value by real-time PCR. BMC Infect Dis.

[26] Cinel G, Pekcan S, Ozçelik U, Alp A, Yalçın E, Doğru Ersöz D (2014). Cytomegalovirus infection in immunocompetent wheezy infants: the diagnostic value of CMV PCR in bronchoalveolar lavage fluid. J Clin Pharm Ther.

[27] Papazian L, Hraiech S, Lehingue S, Roch A, Chiche L, Wiramus S (2015). Cytomegalovirus reactivation in ICU patients. Intensive Care med.

[28] Xue Y, Jiang L, Wan WG, Chen YM, Zhang J, Zhang ZC (2016). Cytomegalovirus pneumonia in patients with rheumatic diseases after immunosuppressive therapy: a single center study in China. Chin med J.

[29] Madi N, Al-Nakib W, Mustafa AS, Saeed T, Pacsa A, Nampoory MR (2007). Detection and monitoring of cytomegalovirus infection in renal transplant patients by quantitative real-time PCR. Med Princ Pract.

[30] Ljungman P, Boeckh M, Hirsch HH, Josephson F, Lundgren J, Nichols G (2017). Definitions of cytomegalovirus infection and disease in transplant patients for use in clinical trials. Clin Infect Dis.

[31] Drew WL (2007). Laboratory diagnosis of cytomegalovirus infection and disease in immunocompromised patients. Curr Opin Infect Dis.

[32] Boeckh M, Stevens-Ayers T, Travi G, Huang ML, Cheng GS, Xie H, et al. Cytomegalovirus (CMV) DNA quantitation in bronchoalveolar lavage fluid from hematopoietic stem cell transplant recipients with CMV pneumonia. J Infect Dis 2017. doi: 10.1093/infdis/jix048. [Epub ahead of print]. 10.1093/infdis/jix048 PMC5461426 28181657

[33] Zhai WJ, Wei JL, Zhao MF, Wang M, Zhou Z, Liu P (2009). Comparison between CMV quantitative PCR and CMV-pp65 antigen test for detection of CMV infection in allogeneic hematopoietic stem cell transplantation. Zhong Guo Shi Yan Xue ye Xue Za Zhi.

[34] Bauer CC, Jaksch P, Aberle SW, Haber H, Lang G, Klepetko W (2007). Relationship between cytomegalovirus DNA load in epithelial lining fluid and plasma of lung transplant recipients and analysis of coinfection with Epstein-Barr virus and human herpesvirus 6 in the lung compartment. J Clin Microbiol.

